# Structural Characterization and Engineering of a GH134 β‑Mannanase from *Aspergillus nidulans* for Enhancement of Activity and Stability

**DOI:** 10.1021/acs.jafc.6c05159

**Published:** 2026-08-27

**Authors:** Sheng-Chia Chen, Po-Chih Kuo, Wen-Ming Chen, Shih-Yi Sheu, Yu-Bin Huang, Cheng-Yang Huang, I-Weh Chien, Tzong-Huei Lee, Chun-Hua Hsu

**Affiliations:** † Department of Agricultural Chemistry, 33561 National Taiwan University, Taipei 106319, Taiwan; ‡ Department of Seafood Science, 517768 National Kaohsiung University of Science and Technology, Kaohsiung 811213, Taiwan; § Department of Marine Biotechnology, 517768 National Kaohsiung University of Science and Technology, Kaohsiung 811213, Taiwan; ∥ Institute of Fisheries Sciences, National Taiwan University, Taipei 106319, Taiwan; ⊥ Department of Life Science, College of Life Science, National Taiwan University, Taipei 106319, Taiwan; # Center for Biotechnology, National Taiwan University, Taipei 106319, Taiwan; ∇ Institute of Biochemical Sciences, National Taiwan University, Taipei 106319, Taiwan; ○ Genome and Systems Biology Degree Program, National Taiwan University and Academia Sinica, Taipei 106319, Taiwan; ◆ Center for Computational and Systems Biology, National Taiwan University, Taipei 106319, Taiwan

**Keywords:** mannanase, GH134, crystallography, protein engineering, CBM

## Abstract

Mannans are abundant plant hemicelluloses, and endo-β-mannanases are important biocatalysts for their conversion into functional manno-oligosaccharides. Here, we report the structural and functional characterization of a glycoside hydrolase family 134 β-mannanase from *Aspergillus nidulans* (AnGH134) and a structure-guided engineering strategy to improve its performance on locust bean gum. The 1.75 Å crystal structure reveals the conserved lysozyme-like fold of GH134 enzymes and supports an inverting catalytic mechanism with Glu43 and Asp55 as the putative catalytic residues. Docking, mutational, and molecular dynamics analyses indicate that AnGH134 uses an extended substrate-binding groove and that groove-exit residues and the C-terminal region contribute to productive catalysis. Guided by these findings, N-terminal fusion of CBM10 enhanced catalytic efficiency and thermal stability, whereas C-terminal fusion was detrimental. These results provide a framework for engineering GH134 mannanases.

## Graphical abstract

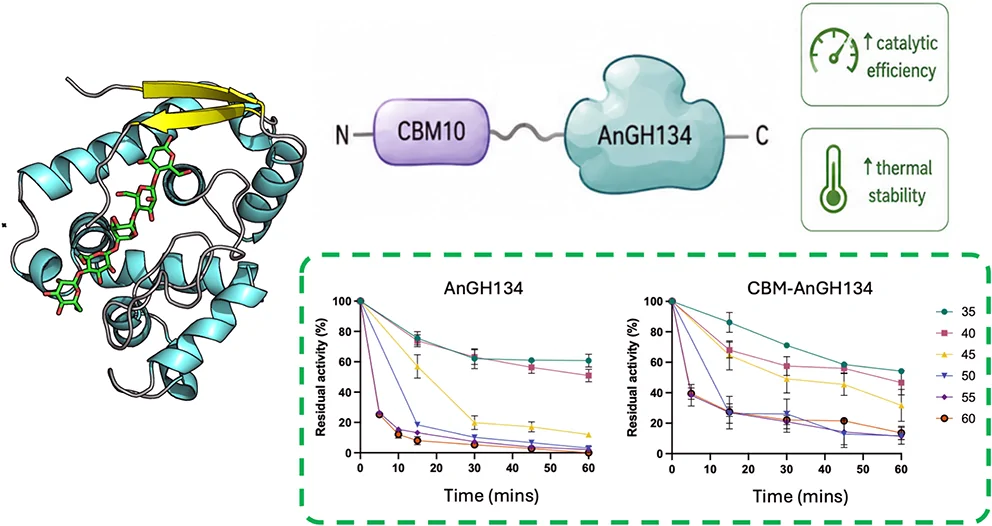

## Introduction

β-Mannans are major constituents of hemicellulose and are widely distributed in plant cell walls.^1,2^ These structurally heterogeneous polysaccharides can be classified into four main types: linear mannans, glucomannans, galactomannans, and galactoglucomannans.^3^ Their backbones are typically composed of β-1,4-linked mannose residues or mixed mannose and glucose residues, and galactomannans and galactoglucomannans are further decorated with α-1,6-linked galactose side chains at the O6 position of mannose.^4^ Enzymatic hydrolysis of mannans produces soluble manno-oligosaccharides that are relevant to agricultural biomass utilization^5^ and useful in functional food production and industrial processing. While mannan-rich hydrocolloids such as locust bean gum are widely used to modulate viscosity and texture in formulated foods, there is a growing demand for transforming these polymers into bioactive manno-oligosaccharides. This creates practical demand for efficient mannan depolymerization under mild conditions. In such processing settings, enzyme dosage and operational stability often dictate economic feasibility.

Endo-β-mannanases are found in multiple glycoside hydrolase (GH) families,^6^ including GH5,^7^ GH26,^8^ GH113,^9^ and GH134.^10^ Enzymes from GH5, GH26, and GH113 typically adopt a (β/α)_8_-barrel fold, whereas GH134 enzymes are distinctive in possessing a lysozyme-like fold and catalyzing hydrolysis with inversion of stereochemistry.^10,11^ Crystal structures of SsGH134 from *Streptomyces sp*. NRRL B-24484 ^10^ and RmGH134 from *Rhizopus microsporus* ^11^ have established a conserved catalytic core and an extended substrate-binding groove in this family. However, the molecular determinants that govern productive binding along the extended groove, as well as the contribution of appended carbohydrate-binding modules (CBMs)^12^ to the balance between catalytic efficiency and stability, remain incompletely understood.^13^ SsGH134 contains a CBM10 that binds insoluble microcrystalline cellulose and β-mannan,^14^ and removal of this CBM was reported to increase catalytic efficiency but compromise stability, highlighting a structure–function trade-off that is directly relevant to enzyme engineering for practical bioprocessing.

In this work, we characterize a GH134 β-mannanase from *Aspergillus nidulans* (AnGH134)^15^ and define the structural and dynamic elements that govern substrate recognition. We combine high-resolution crystallography with docking, mutational analysis of groove residues, and molecular dynamics simulations to examine how an extended binding groove and a ligand-responsive C-terminal tail cooperatively shape productive substrate binding. Guided by this mechanistic framework, we further evaluate a domain-fusion strategy^16,17^ by attaching a CBM10 from *Saccharophagus degradans* (residues 471–507, WP_303492112.1) to either terminus of AnGH134. We show that N-terminal CBM fusion improves catalytic efficiency and thermal stability toward locust bean gum, whereas C-terminal engineering is detrimental, thereby providing design principles for developing GH134 biocatalysts for agricultural and food-related applications.

## Materials and Methods

### Protein Expression and Purification of AnGH134

The *E. coli* codon-optimized gene encoding AnGH134 (residues 19–191, XP_660314.1)^15^ was chemically synthesized (Genomics, Inc., Taiwan) and subcloned into the pET-28a vector (Novagen, Madison, WI, USA) using the *NdeI* and *XhoI* restriction sites. The resulting plasmid was transformed into *E. coli* BL21­(DE3) cells for recombinant expression. To investigate structure–function relationships and generate engineered variants, additional constructs were prepared by PCR-based mutagenesis and cloning. Tyr15 and Trp167 were individually replaced with alanine by site-directed mutagenesis using the primers listed in Table S1, and the double mutant was generated by sequential mutagenesis. In parallel, a C-terminal extension variant and N- or C-terminal CBM (residues 471–507, WP_303492112.1)^18^ fusion constructs were generated by PCR assembly using overlap-containing primers. A serine-rich linker was introduced between AnGH134 and the CBM in the fusion constructs. All constructs were verified by DNA sequencing prior to expression (Table S2).

Cells were grown at 37 °C in Luria–Bertani (LB) medium supplemented with 50 μg mL^–1^ kanamycin. Protein expression was induced at an optical density at 600 nm (OD_600_) of approximately 0.4–0.6 by the addition of 1.0 mM isopropyl β-D-1-thiogalactopyranoside (IPTG), followed by incubation at 25 °C for 20 h. Cells were harvested by centrifugation and resuspended in buffer A (20 mM Tris-HCl, pH 8.0, 100 mM NaCl). The suspension was lysed by sonication, and cell debris was removed by centrifugation at 13,000 rpm for 20 min at 4 °C. The clarified lysate was loaded onto a 3 mL nickel-nitrilotriacetic acid (Ni-NTA) agarose column. The column was washed with buffer A containing 25 mM imidazole, and the target protein was eluted with buffer A containing 250 mM imidazole.

For further purification, the eluate was subjected to size-exclusion chromatography on a Superdex 75 Increase column pre-equilibrated with buffer A. Protein concentration was determined by measuring the absorbance at 280 nm, and purity was assessed by sodium dodecyl sulfate-polyacrylamide gel electrophoresis (SDS-PAGE).^19^ Protein samples were mixed with SDS loading buffer under reducing conditions, heated at 95 °C for 5 min, and separated on 12% resolving polyacrylamide gels. The gels were stained with Coomassie Brilliant Blue R-250 and destained with a methanol/acetic acid solution. The engineered variants were expressed and purified using the same protocol as that used for wild-type AnGH134.

### Enzyme Characterization

AnGH134 activity was measured using the 3,5-dinitrosalicylic acid (DNS) method^20^ with 1% (w/v) locust bean gum (LBG) (Sigma-Aldrich, St. Louis, MO, USA) as the substrate. Reaction mixtures (150 μL) were prepared by mixing substrate in 50 mM McIlvaine buffer (pH 6.0), pre-equilibrated at 40 °C, with 50 μL of appropriately diluted enzyme solution (600 nM). Reactions were incubated at 40 °C for 10 min and terminated by the addition of 150 μL DNS reagent, followed by boiling for 10 min to develop color. Enzyme activity was quantified by measuring the absorbance at 540 nm.

To determine the optimal pH, enzyme assays were performed using McIlvaine buffer (pH 3.0–7.0), Tris-HCl buffer (pH 8.0–9.0), and Gly-NaOH buffer (pH 10.0). The optimal temperature was determined over the range of 10–70 °C in 50 mM McIlvaine buffer (pH 6.0). For thermal stability analysis, enzymes were preincubated at the indicated temperatures for the specified time periods, and residual activity was then measured under the standard assay conditions described above.

The effects of metal ions and chemical agents on enzyme activity were evaluated using the 3,5-dinitrosalicylic acid (DNS) assay. Each additive was individually added to the reaction mixture at a final concentration of 10 mM, and the reactions were incubated at 40 °C for 10 min before measuring enzyme activity. Enzyme activity measured in the absence of metal ions or chemical reagents was defined as the control (100% activity).

To evaluate the effects of organic solvents on mannanase activity, each solvent was added to the reaction system at a final concentration of 10% (v/v), and the reactions were performed at 40 °C for 10 min. Enzyme activity measured without the addition of organic solvents was used as the control.

### Enzyme Kinetics

Apparent kinetic parameters of AnGH134 and CBM-AnGH134 were determined using the DNS assay^20^ with polymeric substrates under identical assay conditions. Standard reactions were performed in a total volume of 250 μL containing 10 μg enzyme and varying concentrations of substrate (ranging from 0 to 10 mg/mL for LBG and 0 to 5 mg/mL for INM) in the appropriate buffer. Reactions were performed at 40 °C for 10 min, and the amount of reducing sugars released was quantified by the DNS method.^20^ The 10 min reaction time was selected within the linear range of product formation under the assay conditions used, as confirmed from the original time-course data at the lowest and highest substrate concentrations tested.

For assays using locust bean gum (LBG), guar gum (GG) or ivory nut mannan (INM), the substrate powder was suspended in water and heated to boiling for 5 min to ensure complete dissolution. The solution was then cooled, centrifuged at 12,000 rpm for 10 min at 10 °C, and the resulting supernatant was used for enzymatic reaction. After incubation, 250 μL DNS reagent was added to terminate the reaction. The mixtures were heated in a boiling water bath for 10 min, cooled to room temperature, and 200 μL aliquots were transferred to a 96-well microplate for absorbance measurement at 540 nm. Reducing sugar concentrations were determined from a mannose standard curve after subtraction of substrate and enzyme blanks.

Apparent kinetic parameters were obtained by fitting the initial rate data to the Michaelis–Menten equation using nonlinear regression. Because LBG, GG and INM are heterogeneous polymeric substrates and reducing sugars were quantified by the DNS method, the kinetic values reported here should be regarded as apparent parameters for relative comparison between constructs rather than absolute catalytic constants.

### High-Performance Anion-Exchange Chromatography with Pulsed Amperometric Detection (HPAEC-PAD)

Hydrolysis products generated by AnGH134 and CBM-AnGH134 from locust bean gum (LBG) and ivory nut mannan (INM) were analyzed using high-performance anion-exchange chromatography with pulsed amperometric detection (HPAEC-PAD). Commercial mannose and manno-oligosaccharide standards (M2-M5) were used as references for retention-time assignment. Analyses were performed using a DIONEX ICS-5000 system (Thermo Fisher Scientific) equipped with a pulsed amperometric detector. Separation was achieved using a CarboPac PA200 analytical column (3 × 250 mm) maintained at 30 °C. Samples (10 μL) were injected and eluted isocratically with 45 mM NaOH at a flow rate of 0.5 mL/min for 25 min.

### Crystallization and Data Collection

Crystallization trials for AnGH134 were performed using the sitting-drop vapor-diffusion method at 10 °C with commercially available screening kits. Crystallization drops were prepared by mixing 2 μL of AnGH134 solution (13 mg mL^–1^) with 2 μL reservoir solution in a 1:1 ratio. Crystals were obtained under reservoir conditions containing 8 mM 1,6-hexanediol, 8 mM 1-butanol, 8 mM 1,2-propanediol, 8 mM 2-propanol, 8 mM 1,4-butanediol, 8 mM 1,3-propanediol, 40 mM imidazole-MES monohydrate (pH 6.1), 9.6% (v/v) precipitant mix (6.4% ethylene glycol and 3.2% (w/v) PEG 8000), and 3.0% (w/v) 1,5-diaminopentane dihydrochloride. For cryoprotection, crystals were transferred to reservoir solution supplemented with 25% (v/v) glycerol and then flash-cooled in liquid nitrogen. X-ray diffraction data were collected at the National Synchrotron Radiation Research Center (NSRRC), Taiwan, on beamline TPS BL05A. Diffraction images were processed, integrated, and scaled using HKL2000.^21^

### Structure Determination

The crystal structure of AnGH134 was determined by the molecular replacement method using Phaser^22^ in the PHENIX package.^23^ The coordinate of SsGH134 (PDB code: 5JU9) was used as the search model. The initial structure was refined through iterative cycles of simulated annealing, energy minimization, and manual rebuilding using PHENIX refinement^24^ and COOT.^25^ Molecular visualizations were generated with PyMOL (The PyMOL Molecular Graphics System, Schrödinger, LLC). Data collection and refinement statistics are summarized in Table 1.

**Table 1:** X-ray Data Collection and Refinement Statistics^a^

Data collection	AnGH134
Wavelength (Å)	0.9998
Space group	*C*2
Unit cell (Å)	*a* = 115.9, *b* = 50.2, *c* = 67.7, β = 105.9
Resolution (Å)	30.00–1.75 (1.81–1.75)
Total observations	139741 (12054)
Unique reflections	37139 (3258)
Redundancy	3.7 (3.7)
R_merge_ (%)^b^	8.3 (52.8)
Completeness (%)	99.8 (100.0)
*I/σ (I)*	14.7 (2.3)
Refinement	
No. of unique reflections used in refinement	37137
No. of reflections in R_free_ test set	2000
R_work_ (%)^c^	15.9 (22.9)
R_free_ (%)^c^	18.6 (27.1)
r.m.s.d.	
Bond lengths (Å)	0.006
Bond angles (°)	0.71
Ramachandran plot (%)	
Most favored	98.2
Allowed	1.8
Average B-values (Å^2^)	
Protein	22.6
Ligands	36.5
Water	37.7
PDB code	24MP

a Values in parentheses show the statistics for the highest resolution shells.

b R_merge_= Σ_hkl_[(Σ_i_|I_i_-_i_>|)/Σ_i_|I_i_|].

c R_work_= (Σ_hkl_||F_o_| - |F_c_||)/Σ_hkl_|F_o_|. R_free_was calculated with 5% of the data excluded from refinement.

### Circular Dichroism (CD) Spectroscopy

Far-UV circular dichroism (CD) spectra of wild-type AnGH134 and its variants were recorded using a JASCO J-815 spectrometer with 10 μM protein samples in 20 mM phosphate buffer. Samples were placed in a 1 mm path-length cuvette, and spectra were collected under the indicated conditions. For thermal denaturation experiments, the CD signal was monitored at 209 nm from 10 to 95 °C at a scan rate of 1 °C/min. The apparent melting behavior was analyzed using the first derivative of the CD signal. Under the conditions used, Y15A and the Y15A/W167A double mutant did not exhibit typical sigmoidal unfolding transitions.

### Differential Scanning Fluorimetry (DSF)

DSF experiments were performed using a StepOne Real-Time PCR Detection System (Thermo Fisher). Each 25 μL reaction mixture contained 2 μL of SYPRO Orange (Sigma-Aldrich) and 10 μM protein sample in 20 mM phosphate buffer. Fluorescence signals were recorded from 10 to 95 °C (excitation, 450–490 nm; detection, 560–580 nm). Data evaluation and Tm determination were carried out using SigmaPlot. For the Y15A/W167A double mutant, no typical sigmoidal unfolding curve was observed under the conditions used, and therefore a reliable Tm value could not be determined by DSF. All measurements were performed in triplicate.

### Label-Free Differential Scanning Fluorimetry (nanoDSF)

NanoDSF experiments were performed using a Tycho NT.6 instrument (NanoTemper Technologies). Protein samples (10 μM) in 20 mM phosphate buffer were loaded into glass capillaries and heated from 35 to 95 °C at a rate of 30 K/min, while intrinsic fluorescence at 330 and 350 nm was recorded. Data analysis, data smoothing, and derivative calculations were carried out using the internal evaluation features of the Tycho instrument. Apparent Tm values were determined from the first derivative of the fluorescence ratio (350 nm/330 nm).

### Molecular Dynamics Simulations

Molecular dynamics (MD) simulations were performed using GROMACS 2020.6 ^26^ with the CHARMM36m all-atom force field for the protein^27^ and the TIP3P water model for explicit solvation.^28^ For the ligand-bound model, the coordinates of mannopentaose (M5) were generated by structure-guided transfer from a reference SsGH134/M5 complex (PDB ID: 5JUG)^10^ after superposition of the protein backbones. The topology and parameters for M5 were generated using the CHARMM-compatible carbohydrate topology file (CARA.itp) prepared for the ligand.

Each system was placed in a dodecahedral simulation box with a minimum distance of 12 Å between the protein and the box boundary, followed by solvation with explicit water molecules. Na^+^ and Cl^–^ ions were then added to neutralize the system and achieve a final ionic strength of 150 mM. The apo system contained 31 Na^+^, 26 Cl^–^, and 9267 water molecules, whereas the ligand-bound AnGH134/M5 system contained 31 Na^+^, 26 Cl^–^, and 9276 water molecules.

Energy minimization was carried out using the steepest descent algorithm until the maximum force was below 1000 kJ mol^–1^ nm^–1^. Each system was then equilibrated in two successive steps: 100 ps under NVT conditions at 300 K, followed by 1 ns under NPT conditions at 300 K and 1 bar. Temperature was maintained using the V-rescale thermostat,^29^ and pressure was controlled with the Parrinello–Rahman barostat.^30^ All covalent bonds involving hydrogen atoms were constrained using LINCS,^31^ allowing a 2 fs integration time step. Long-range electrostatic interactions were calculated using the particle mesh Ewald (PME) method.^32^

For each system, three independent MD simulations were performed. Each replicate was independently subjected to energy minimization, NVT equilibration, NPT equilibration, and a 200 ns production run in the NPT ensemble using a different initial velocity seed. Trajectory coordinates were recorded every 2 ps for subsequent analysis. Trajectory analysis was conducted using built-in GROMACS utilities. Structural stability was evaluated by backbone root-mean-square deviation (RMSD) analysis using gmx rms, and residue-level flexibility was assessed by root-mean-square fluctuation (RMSF) analysis using gmx rmsf.

## Results

### Overall Structure of AnGH134

The amino acid sequence of AnGH134 was obtained from UniProt entry Q5B9S0. Signal peptide prediction using SignalP 5.0 server^33^ indicated that residues 1–18 constitute a putative N-terminal signal peptide. Accordingly, the DNA fragment encoding residues 19–191, corresponding to the predicted mature form of AnGH134, was chemically synthesized and used for recombinant protein production. After expression and purification, the mature AnGH134 protein was successfully crystallized. SDS-PAGE analysis of the purified protein and photographs of the crystals are shown (Figure S1).

The crystal structure of AnGH134 was determined at 1.75 Å resolution in the C2 space group, with two molecules in the asymmetric unit (Figure 1A). Despite the presence of two molecules in the crystal lattice, size-exclusion chromatography indicated that AnGH134 exists as a monomer in solution (Figure 1B), suggesting that the crystallographic dimer does not represent the predominant solution state under the assay conditions. The apparent molecular weight estimated from the elution volume, approximately 9.8 kDa, is smaller than the theoretical molecular weight of AnGH134 (19.4 kDa). This discrepancy likely reflects nonideal behavior during size-exclusion chromatography. As a carbohydrate-active enzyme with an extended substrate-binding groove for polymeric mannans, AnGH134 may exhibit weak interactions with the polysaccharide-based matrix of the column or adopt a compact hydrodynamic shape, either of which could lead to underestimation of its apparent molecular size.

**Figure 1:**
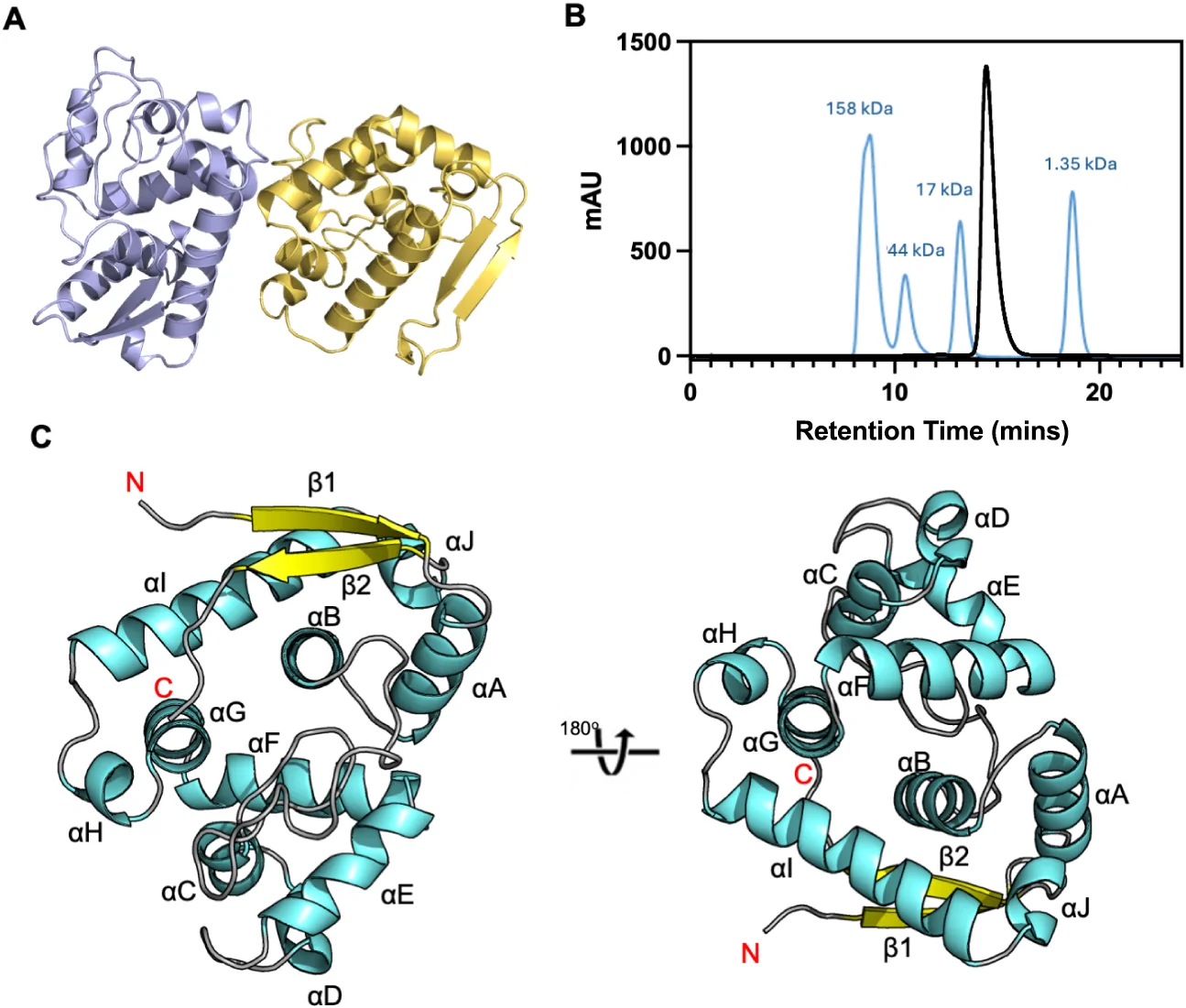
Overall structure of AnGH134. (A) Crystal structure of AnGH134 determined at 1.75 Å resolution. Two molecules are present in the asymmetric unit of the C2 crystal form. (B) Size-exclusion chromatography profile of purified AnGH134, indicating that the enzyme behaves as a monomer in solution under the assay conditions. (C) Overall ribbon representation of AnGH134 showing the characteristic lysozyme-like fold of GH134 enzymes, composed of ten α-helices (αA-αJ) and two antiparallel β-strands (β1−β2). The catalytic residues Glu43 and Asp55 are shown as sticks.

AnGH134 adopts the characteristic lysozyme-like fold of GH134 enzymes, comprising ten α-helices (αA-αJ) and two antiparallel β-strands (β1-β2) (Figure 1C). The overall architecture forms an elongated substrate-binding groove that extends across the protein surface (Figure 2A), consistent with an endoacting mode of hydrolysis toward polymeric mannan substrates. The hydrolytic behavior of AnGH134 is therefore expected to resemble that of other GH134 β-mannanases, including RmGH134 ^11^ and SsGH134.^14^ Structural superposition with the SsGH134 revealed strong conservation of the catalytic core and positioned Glu43 and Asp55 at the center of the groove (Figure 2B). By analogy with SsGH134, these residues are assigned as the putative general acid (Glu43) and general base (Asp55), supporting an inverting catalytic mechanism. Collectively, the high-resolution structure, together with the monomeric solution behavior, provides a structural basis for interpreting substrate recognition and guiding subsequent mutational and engineering analyses.

**Figure 2:**
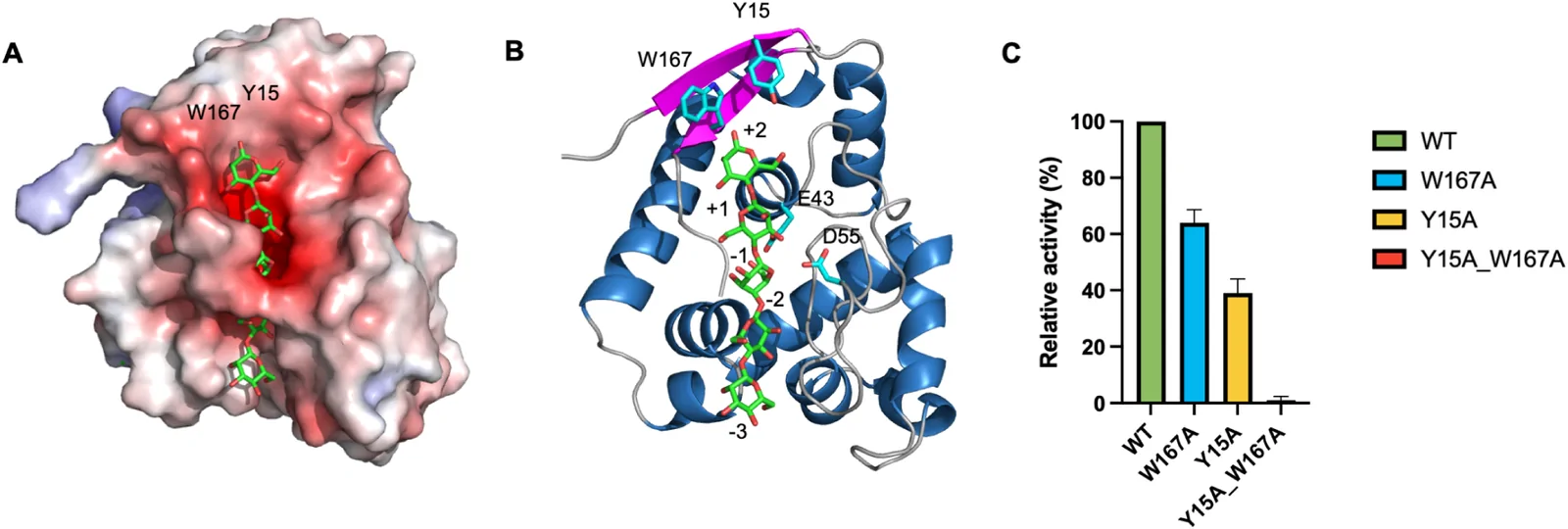
Structural model of substrate binding and mutational analysis of groove-exit residues in AnGH134. (A) Structural model of the AnGH134-M5 complex generated by structure-guided ligand transfer. The M5 ligand is shown as sticks within the elongated substrate-binding groove of AnGH134. (B) Surface representation of the AnGH134-M5 model showing the electrostatic environment of the substrate-binding groove. The bound pentamannose spans subsites −3 to +2. Residues Tyr15 and Trp167, located near the distal side of the +2 subsite, form a previously uncharacterized gate-like barrier at the groove exit and are highlighted. (C) Relative activities of wild-type AnGH134 and groove-exit mutants (Y15A, W167A, and W167A/Y15A) toward locust bean gum (LBG). Both single mutants showed reduced activity, whereas the double mutant exhibited no detectable activity, supporting the functional importance of these residues in substrate engagement and productive turnover of polymeric mannan substrates. Data are shown as mean ± SD.

To model substrate recognition, we generated an AnGH134-M5 complex using a structure-guided ligand transfer approach. Specifically, AnGH134 was superimposed onto a reference GH134 complex structure (PDB ID: 5JUG), and the coordinates of the bound pentamannose (M5) ligand were transferred into the active-site groove of AnGH134 (Figure 2A). The resulting model places M5 within the deep substrate-binding groove in a catalytically plausible orientation (Figure 2B). Inspection of the model suggests that the catalytic cleft contains multiple sugar-binding subsites, with the pentamannose (M5) spanning subsites −3 to +2 along the groove.

Interestingly, the AnGH134-M5 model revealed a previously uncharacterized structural feature at the distal side of the +2 subsite, where Tyr15 and Trp167 appear to form a gate-like barrier at the groove exit (Figure 2B). Structurally, Tyr15 and Trp167 are located on the β1 and β2 strands, respectively, which together frame this distal groove region. This arrangement suggests that the two residues may help confine or position extended mannan chains within the binding groove and thereby support productive catalysis on polymeric substrates. The primary goal of the mutagenesis was therefore to test whether these previously uncharacterized “groove-edge” residues contribute to the productive turnover of polymeric substrates. In particular, Trp167 is highly conserved within the GH134 family, further supporting its potential functional importance. To examine this possibility, Y15A and W167A single mutants, as well as the Y15A/W167A double mutant, were generated. Both single mutants showed reduced activity, whereas the double mutant exhibited no detectable activity (Figure 2C). Together, these results suggest that Tyr15 and Trp167 contribute to efficient processing of extended galactomannan substrates such as LBG, likely through roles in groove-edge substrate engagement and maintenance of a functionally competent groove architecture.

To further assess whether the reduced activities of the mutants might also involve altered structural stability, the variants were analyzed by DSF, nanoDSF, and far-UV CD spectroscopy (Figure S2). In DSF, the Tm values of the wild-type, Y15A, and W167A proteins were 45.4 ± 0.06 °C, 44.06 ± 0.07 °C, and 46.27 ± 0.03 °C, respectively, whereas a reliable Tm value could not be determined for the Y15A/W167A double mutant because no typical sigmoidal unfolding transition was observed. In nanoDSF, the corresponding Tm values were 55.7 °C, 53.1 °C, 55.8 °C, and 54.6 °C for the wild-type, Y15A, W167A, and Y15A/W167A proteins, respectively. These results indicate that the single mutants show only modest changes in thermal stability relative to the wild-type enzyme.

In addition, the far-UV CD spectrum of the double mutant differed markedly from that of the wild-type enzyme, and CD-monitored thermal denaturation at 209 nm showed that Y15A and the Y15A/W167A double mutant did not exhibit typical sigmoidal unfolding behavior under the conditions tested. Together, these observations suggest that some conformational perturbations are not fully captured by Tm values alone. Because Tyr15 and Trp167 are located on the β1 and β2 strands that frame the groove exit, simultaneous substitution of these residues may perturb not only groove-edge substrate interactions but also the local structural framework of this gate-like region. Thus, the complete loss of activity in the double mutant may reflect combined effects on substrate engagement and local structural integrity.

### Molecular Simulation of AnGH134 in Apo and Complex Forms

Because an experimental crystal structure of the AnGH134-M5 complex could not be obtained, molecular dynamics (MD) simulations were conducted to investigate the stability and conformational behavior of the modeled complex, with particular emphasis on the C-terminal region. Two systems were analyzed, including apo AnGH134 and the AnGH134-M5 complex, and each system was simulated for 200 ns. In both cases, the protein remained monomeric throughout the simulation. All simulations were performed in explicit solvent at 300 K in the presence of 150 mM NaCl to approximate the ionic conditions used in the biochemical experiments.

Backbone RMSD analysis showed that both systems gradually converged and reached stable plateaus over the course of the simulations, indicating that the trajectories were overall well equilibrated (Figure 3A). The AnGH134-M5 complex maintained relatively low RMSD values after equilibration, supporting the structural stability of the modeled ligand-bound state and suggesting that M5 binding does not induce major global conformational changes. RMSF analysis further showed that most regions of the protein exhibited only moderate flexibility (Figure 3B). In the apo simulation, however, the C-terminal segment spanning residues 168–173 displayed substantially greater mobility, indicating increased conformational disorder in the absence of substrate. This elevated flexibility was markedly reduced in the ligand-bound simulation, consistent with ligand-associated stabilization of the C-terminal region.

**Figure 3:**
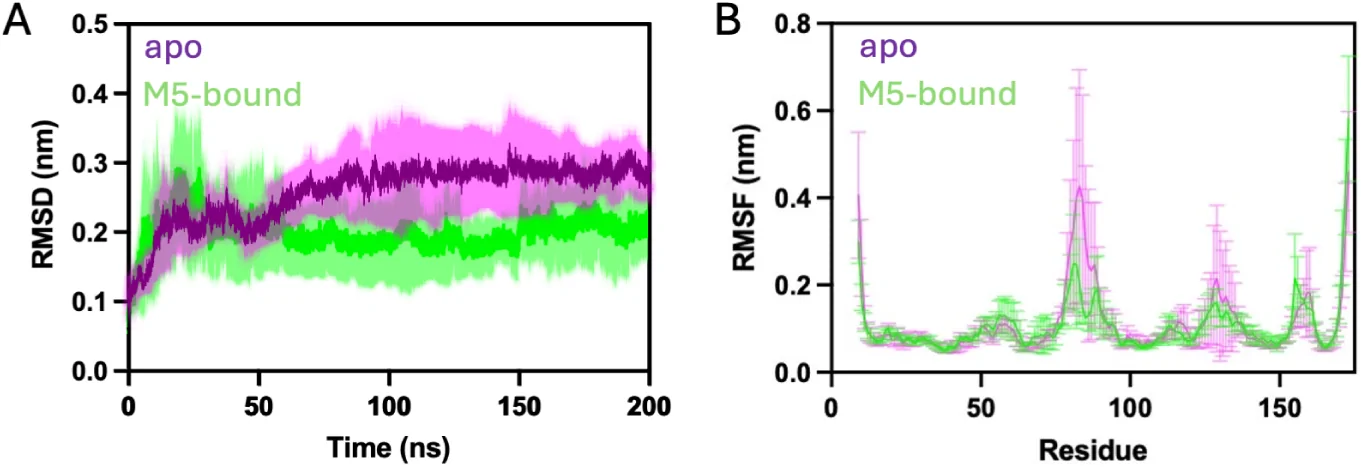
Molecular dynamics simulations of AnGH134 in apo and ligand-bound states. (A) Backbone root-mean-square deviation (RMSD) profiles of apo AnGH134 and the AnGH134-M5 complex over 200 ns MD simulations, showing that both systems reached stable plateaus during the simulation period. (B) Per-residue root-mean-square fluctuation (RMSF) profiles of apo AnGH134 and the AnGH134-M5 complex. The C-terminal segment (residues 168–173) displayed elevated mobility in the apo state but reduced fluctuation in the ligand-bound state, indicating ligand-associated stabilization of the C-terminal region.

Notably, the region spanning residues 80–96 also exhibited high conformational flexibility in the apo state. This structural plasticity may help maintain a more open active-site environment that could facilitate the entry of carbohydrate polymers. In the AnGH134-M5 simulation, however, this region became markedly less flexible upon ligand binding, even though it does not directly contact M5. These observations are consistent with the possibility that the 80–96 segment acts as a secondary-shell element of the binding pocket and becomes more ordered as the groove adopts a ligand-stabilized conformation. By contrast, residues 150–165 exhibited increased fluctuations in the ligand-bound state, possibly reflecting coupled motions associated with the opposing C-terminal β-strand located near the modeled M5-binding region. Taken together, these results support a model in which substrate binding is associated with local rearrangement and ordering around the binding groove, while restraining the motion of the C-terminal tail. These observations are consistent with a potential role for these regions in ligand-responsive stabilization during catalysis.

### Protein Engineering of AnGH134

Guided by the MD simulations, we hypothesized that the C-terminal region of AnGH134 contributes to productive interactions with M5 and may therefore influence catalytic performance. To test whether extension of the C-terminal region could further stabilize interactions with polymeric substrates, we introduced a short six-residue C-terminal extension (VDVQAI) (Figure 4A). This design was based on structural considerations: the terminal six residues of the C-terminal region are not well ordered in the apo form, whereas in the M5-bound model this region becomes more ordered and is positioned to form hydrogen-bond interactions near the distal part of the substrate-binding groove. We therefore hypothesized that modest extension of this ligand-responsive region might influence mannan binding and stabilization at the groove exit. Contrary to our expectation, this modification completely abolished detectable enzymatic activity. These results suggest that the native length and conformational positioning of the C-terminal region are tightly constrained in GH134 enzymes and that even a short extension is sufficient to disrupt the geometry required for carbohydrate binding and catalysis.

**Figure 4:**
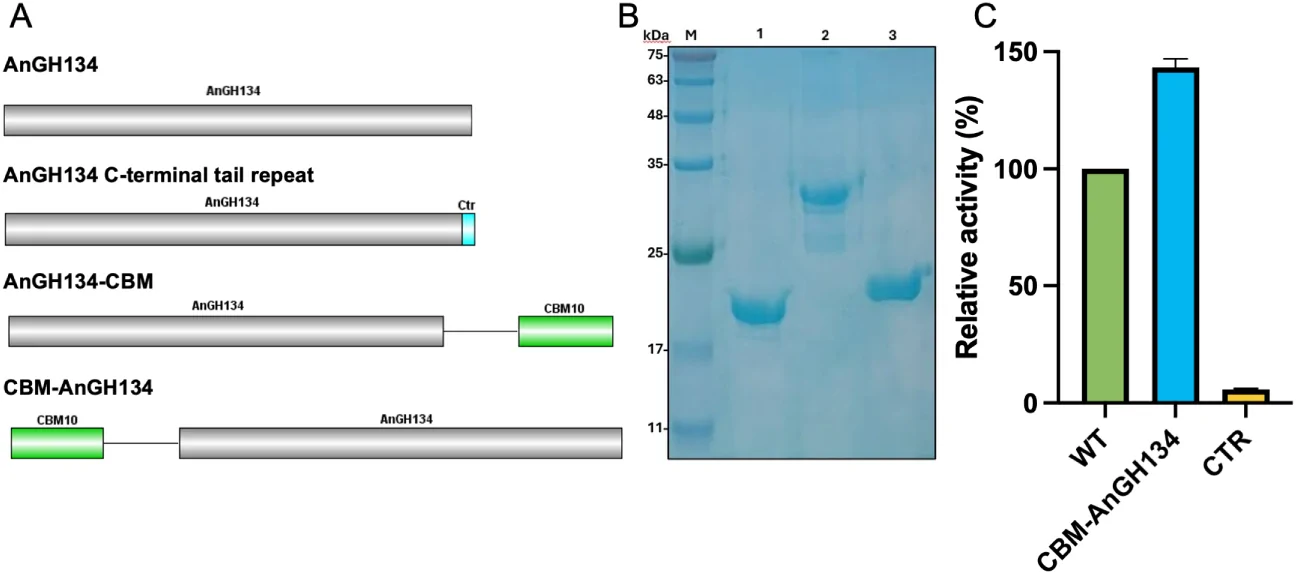
Protein engineering of AnGH134 based on structural and dynamic analyses. (A) Schematic representation of wild-type AnGH134 and engineered variants. Variants include the C-terminal extension construct (CTR), the N-terminal CBM10 fusion construct (CBM-AnGH134), and the C-terminal CBM10 fusion construct (AnGH134-CBM). A serine-rich linker was introduced between the catalytic domain and CBM10 in the fusion constructs. (B) SDS-PAGE analysis of purified recombinant proteins. Wild-type AnGH134, the CTR variant, and CBM-AnGH134 were successfully expressed and purified. The C-terminal fusion construct (AnGH134-CBM) was not recovered as a soluble purified protein under the expression conditions used. (Lane M, protein marker; lane 1, wild-type AnGH134; lane 2, CBM-AnGH134; lane 3, CTR variant.) (C) Relative activities of wild-type AnGH134 and engineered variants toward LBG. The C-terminal extension abolished detectable activity, whereas N-terminal fusion of CBM10 enhanced activity relative to the wild-type enzyme. Data are shown as mean ± SD.

To further improve enzymatic performance, we next explored a domain-fusion strategy by attaching the carbohydrate-binding module CBM10 from *Saccharophagus degradans* to either the N terminus or the C terminus of AnGH134 (Figure 4A). The N-terminal fusion was successfully expressed and purified (Figure 4B). In contrast, the C-terminal fusion construct (AnGH134-CBM) was expressed predominantly in the insoluble fraction (inclusion body) under the expression conditions used, and no corresponding target protein was recovered from the soluble fraction during purification (Figure S3). Therefore, this construct could not be characterized further. We therefore compared the activity of the wild-type enzyme with that of the N-terminal fusion construct, CBM-AnGH134. Under identical assay conditions, CBM-AnGH134 exhibited higher activity than the wild-type enzyme (Figure 4C), indicating that N-terminal CBM fusion is an effective strategy for enhancing enzymatic performance.

Mannanase activities of AnGH134 and CBM-AnGH134 were determined using the DNS assay with locust bean gum (LBG) as the substrate. Both enzymes showed maximal activity in 50 mM sodium citrate-phosphate buffer at pH 6.0, and the optimal temperature for both constructs was 40 °C (Figure 5A). For both enzymes, activity declined sharply at temperatures above 50 °C. These values are broadly consistent with those reported in the initial biochemical characterization of AnGH134 ^15^, indicating that the recombinant enzyme used in the present study retains the expected catalytic properties. In this context, the main additional insights of the present work arise not from redefinition of the basic biochemical optima, but from the integration of crystal structure determination, substrate-binding analysis, mutagenesis, molecular dynamics simulations, and structure-guided engineering.

**Figure 5:**
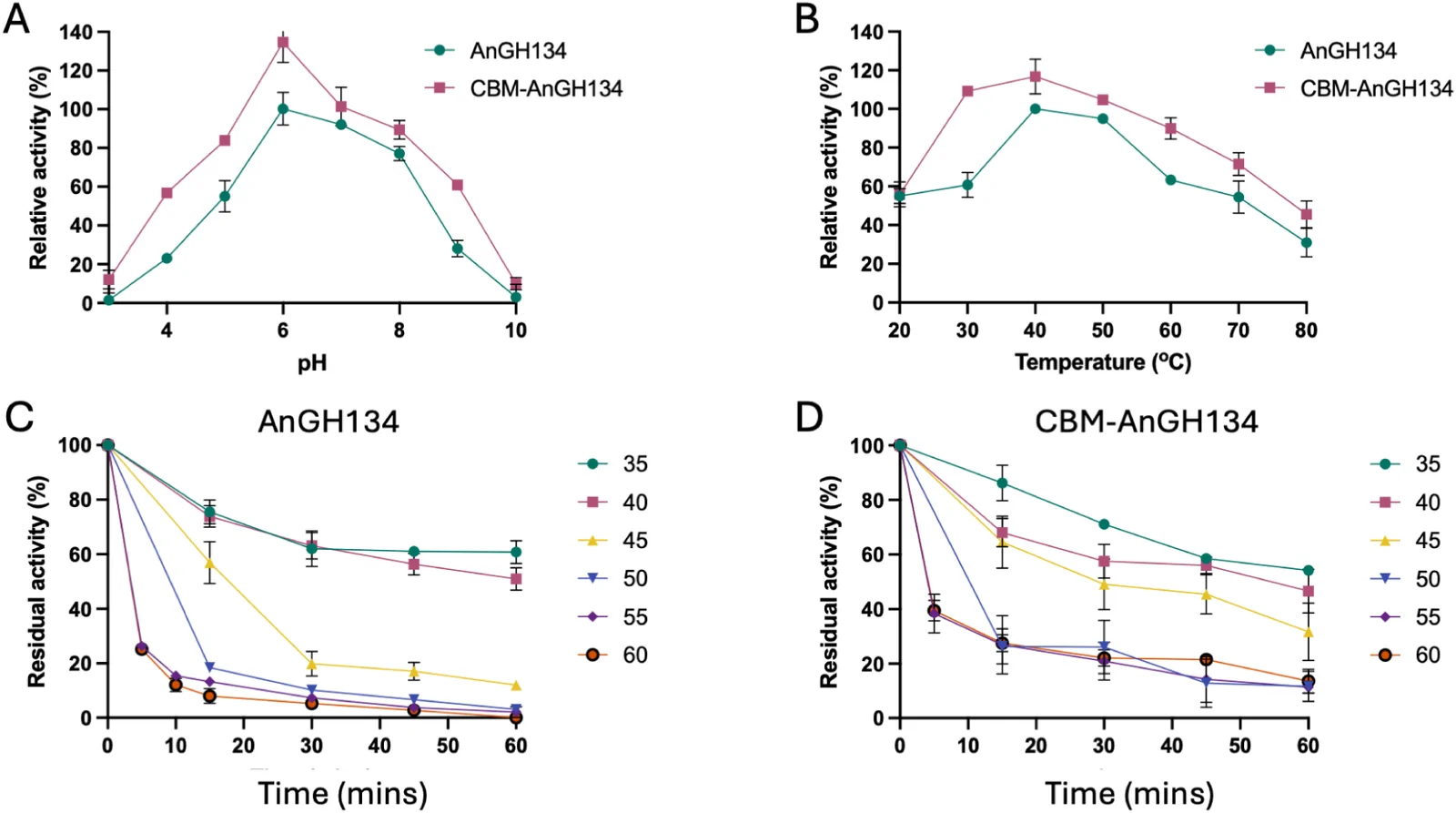
Effects of pH, temperature, and thermal stability on the activities of AnGH134 and CBM-AnGH134. (A) Effect of pH on the activities of AnGH134 and CBM-AnGH134, determined using locust bean gum (LBG) as the substrate. Both enzymes showed maximal activity in 50 mM sodium citrate-phosphate buffer at pH 6.0. (B) Effect of temperature on the activities of AnGH134 and CBM-AnGH134. Both enzymes showed maximal activity at 40 °C. (C) Thermal stability of AnGH134 after incubation at the indicated temperatures for different time intervals. Residual activity is expressed relative to the corresponding activity measured before incubation, which was defined as 100%. (D) Thermal stability of CBM-AnGH134 after incubation at the indicated temperatures for different time intervals. Residual activity is expressed relative to the corresponding activity measured before incubation, which was defined as 100%. Data are shown as mean ± SD.

Thermal stability assays showed that both AnGH134 and CBM-AnGH134 retained more than 60% of their initial activity after incubation at 40 °C for 1 h (Figure 5B). However, AnGH134 showed a pronounced loss of activity after exposure to temperatures above 45 °C, indicating greater susceptibility to thermal inactivation (Figure 5C). In contrast, CBM-AnGH134 retained higher residual activity at elevated temperatures (Figure 5D), suggesting that CBM fusion improves resistance to thermal stress and broadens the operational temperature range. Taken together, these results indicate that AnGH134 is best suited for processes conducted at or below 40 °C, whereas CBM-AnGH134 offers improved robustness under moderately elevated temperature conditions.

Kinetic analyses further underscored the beneficial effect of CBM fusion (Table 2). For LBG, AnGH134 exhibited a Km of 1.63 mg mL^–1^ and a kcat/Km of 53.52 mL mg^–1^ s^–1^. In contrast, CBM-AnGH134 showed a lower Km of 1.11 mg mL^–1^ and a markedly higher catalytic efficiency, with a kcat/Km of 126.78 mL mg^–1^ s^–1^ (Table 2; Figure S4A and S4B). For INM, AnGH134 displayed a Km of 6.31 mg mL^–1^ and a kcat/Km of 11.38 mL mg^–1^ s^–1^, whereas CBM-AnGH134 showed a lower Km of 2.47 mg mL^–1^ and a substantially higher catalytic efficiency, with a kcat/Km of 47.45 mL mg^–1^ s^–1^ (Table 2; Figure S4C and S4D). For guar gum (GG), no detectable activity was observed under the assay conditions used, possibly reflecting the high degree of galactose substitution and limited productive hydrolysis under the present assay setup; therefore, apparent kinetic parameters could not be determined. These kinetic data indicate that N-terminal CBM10 fusion improves the apparent substrate affinity of AnGH134 and enhances catalytic turnover in the polymer-based assay. Collectively, these results demonstrate that N-terminal CBM10 fusion is an effective engineering strategy for improving both the catalytic efficiency and thermal robustness of AnGH134 toward LBG, whereas modifications at the C-terminus are poorly tolerated. This observation is consistent with the dynamic and mechanistic importance of the C-terminal region inferred from the MD simulations.

**Table 2:** Apparent Kinetic Parameters of Recombinant AnGH134 and CBM-AnGH134 toward LBG, GG and INM^a^

Enzyme	Substrate	K_m_ (mg.mL^–1^)	k_cat_ (s^–1^)	k_cat_/K_m_ (s^–1^ mg^–1^ mL)
AnGH134	LBG	1.63	87.24	53.52
CBM-AnGH134	LBG	1.11	140.72	126.78
AnGH134	GG	N.D.	N.D.	N.D.
CBM-AnGH134	GG	N.D.	N.D.	N.D.
AnGH134	INM	6.31	71.74	11.38
CBM-AnGH134	INM	2.47	117.11	47.45

a N.D, no detectable activity under the assay conditions used.

To further characterize the hydrolytic products generated from representative β-mannan substrates, reaction mixtures were analyzed by HPAEC (Figure 6). Both AnGH134 and CBM-AnGH134 produced comparable manno-oligosaccharide profiles from LBG and INM under the assay conditions used, providing direct product-level evidence for productive hydrolysis of these β-mannan substrates. The similar product distributions further indicate that N-terminal CBM10 fusion does not substantially alter the overall cleavage pattern of AnGH134. Together with the kinetic data, these results support the ability of AnGH134 and its engineered variant to process β-mannan-based polysaccharides, although broader validation using more complex agricultural biomass substrates will require future investigation.

**Figure 6:**
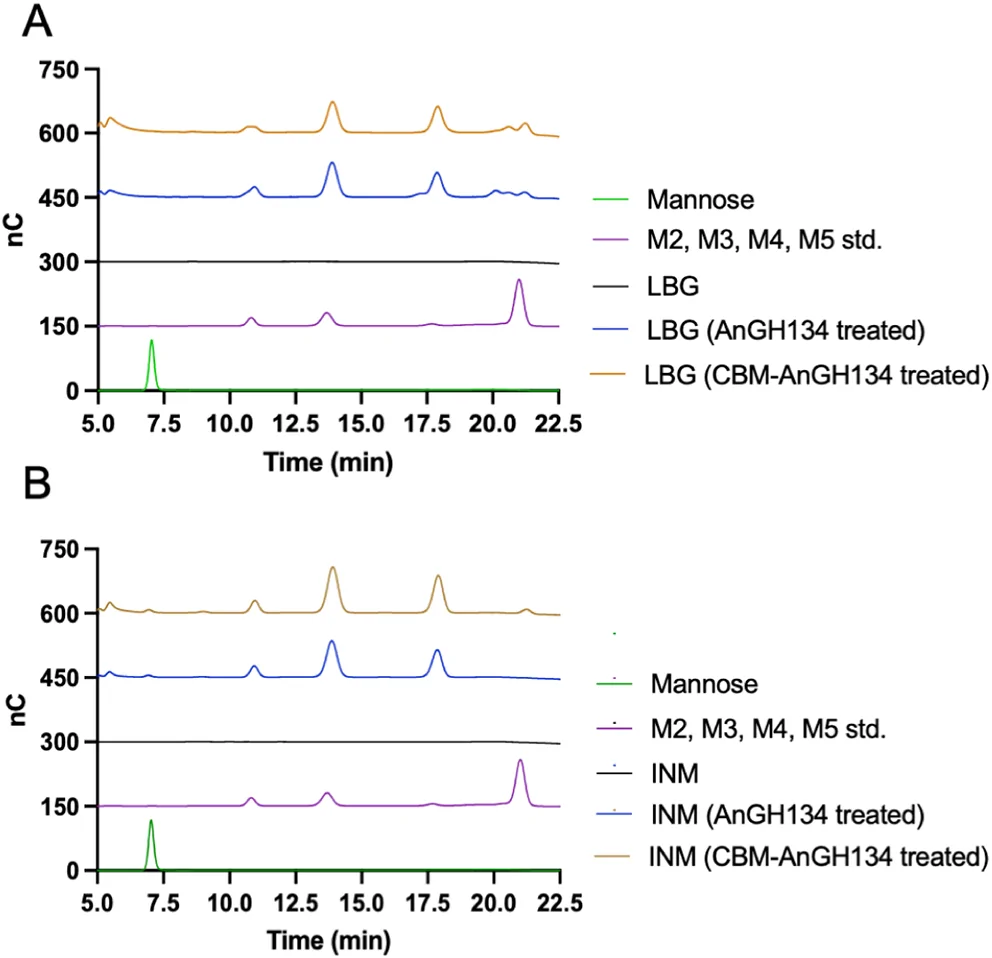
HPAEC analysis of hydrolysis products generated by AnGH134 and CBM-AnGH134 from representative β-mannan substrates. (A) HPAEC-PAD profiles of hydrolysis products generated from locust bean gum (LBG) by AnGH134 and CBM-AnGH134. (B) HPAEC-PAD profiles of hydrolysis products generated from ivory nut mannan (INM) by AnGH134 and CBM-AnGH134. Mannose and manno-oligosaccharide standards (M2-M5) were used for retention-time assignment. Both enzymes generated comparable manno-oligosaccharide product profiles under the assay conditions used.

### Metal Ion Tolerance

The effects of metal ions and chemical agents on enzyme activity were further evaluated. As summarized in Table 3, the addition of metal ions generally reduced the activities of both AnGH134 and CBM-AnGH134, although the extent of inhibition varied among the tested ions. Among them, Cu^2+^ exerted the strongest inhibitory effect, decreasing the relative activities of AnGH134 and CBM-AnGH134 to 34.51% and 41.03%, respectively. Monovalent cations, including Na^+^, K^+^, and Li^+^, also reduced enzyme activity to varying degrees, with Na+ showing a comparatively stronger inhibitory effect on AnGH134. In contrast, divalent cations such as Ca^2+^ and Mg^2+^ were better tolerated, as both enzymes retained most of their activity in their presence. Notably, Zn^2+^ affected the two enzymes differently: whereas it slightly reduced the activity of AnGH134, CBM-AnGH134 retained full activity and even showed a modest increase to 107.07%, suggesting that CBM fusion may confer greater tolerance to this ion.

**Table 3:** Effects of Various Metal Ions, Chemical Agents and Organic Solvents on the Activities of AnGH134 and CBM-AnGH134

		Relative activity (%)	
		AnGH134	CBM-AnGH134^a^
	Control	100	119.19 ± 5.12
Metal ions (10 mM)	Na^+^	61.95 ± 6.40	88.25 ± 7.47
	K^+^	74.74 ± 4.65	86.99 ± 2.06
	Li^+^	80.30 ± 9.51	86.36 ± 4.78
	Ca^2+^	89.64 ± 8.87	98.98 ± 15.47
	Cu^2+^	34.51 ± 8.52	41.03 ± 12.50
	Mg^2+^	82.92 ± 6.14	89.39 ± 2.64
	Ni^2+^	76.93 ± 3.74	82.19 ± 4.56
	Zn^2+^	82.92 ± 6.14	107.07 ± 5.60
Chemical agent (10%)	MeOH	114.47 ± 10.85	129.46 ± 6.93
	EtOH	87.03 ± 10.37	119.19 ± 1.73
	EDTA	80.13 ± 6.81	83.33 ± 8.38
	PEG 6000	109.59 ± 7.77	101.01 ± 15.29
	Tween 20	78.28 ± 4.32	112.12 ± 0.43

a CBM-AnGH134 values are expressed relative to wild-type control.

The effects of chemical agents were more variable. MeOH slightly enhanced the activities of both enzymes, whereas PEG 6000 had little overall effect. EtOH, EDTA, and Tween 20 reduced the activity of AnGH134 to different extents, while CBM-AnGH134 generally showed greater tolerance under the same conditions. In particular, CBM-AnGH134 retained near-complete or even enhanced activity in the presence of EtOH and Tween 20, indicating that CBM fusion may improve enzyme robustness under chemically perturbed conditions.

Collectively, these results demonstrate that N-terminal CBM10 fusion is an effective engineering strategy for enhancing both the catalytic efficiency and thermal robustness of AnGH134 toward LBG, whereas modifications at the C-terminus are poorly tolerated. This observation is consistent with the dynamic and mechanistic importance of the C-terminal region inferred from the MD simulations.

## Discussion

The high-resolution crystal structure of AnGH134 confirms the conserved lysozyme-like fold of GH134 mannanases and supports an inverting catalytic mechanism, with Glu43 and Asp55 positioned as the putative catalytic acid and base residues. Beyond defining the structural framework of AnGH134, our results provide insight into how extended groove interactions, ligand-responsive conformational dynamics, and domain architecture collectively influence catalytic performance and engineering tolerance in GH134 enzymes. From an application perspective, AnGH134 exhibited optimal activity at pH 6.0 and 40 °C toward locust bean gum, a food-grade galactomannan widely used as a viscosity-modifying hydrocolloid. These results support the potential relevance of GH134 enzymes for processing formulated mannans under mild conditions, although further validation using more complex food or agricultural biomass substrates will be needed.

The modeled AnGH134-M5 complex suggests that the substrate is accommodated along an extended groove spanning subsites −3 to +2. Mutational analysis further showed that Trp167 and Tyr15, located near the groove exit, are important for efficient hydrolysis of polymeric substrates. Although these residues lie beyond the core region occupied by the docked pentasaccharide, their pronounced effects on activity indicate that productive turnover of long-chain galactomannans depends on interactions extending beyond the minimal binding register captured by the M5 model. This observation highlights the functional importance of groove-edge architecture in GH134 catalysis and suggests that substrate processing depends not only on the catalytic residues within the central cleft but also on peripheral residues that help guide or stabilize extended polysaccharide chains during turnover.

MD simulations further revealed that AnGH134 contains ligand-responsive flexible regions that may contribute to substrate engagement. In the apo state, both the 80–96 segment and the C-terminal tail displayed elevated mobility, whereas ligand binding reduced the flexibility of these regions. The high conformational plasticity of residues 80–96 in the apo form may help maintain a more open binding groove that could facilitate polymer entry, while its reduced flexibility in the ligand-bound state is consistent with this segment acting as a secondary-shell element that becomes more ordered upon substrate engagement. Similarly, reduced mobility of the C-terminal tail in the AnGH134-M5 simulation is consistent with a role for this region in productive substrate binding and/or groove shaping during catalysis. This interpretation is further supported by the structural snapshots (Figure S5), which reveal more pronounced conformational deviation between the initial and final states in the apo simulation than in the ligand-bound complex. Together, these observations support a model in which substrate recognition is associated with local conformational ordering around the active-site groove rather than with major global structural rearrangements.

This dynamic framework also provides a mechanistic explanation for the engineering outcomes observed in this study. Extension of the C terminus by six residues completely abolished activity, indicating that the native length and positioning of this region are tightly constrained. Likewise, the inability to obtain soluble protein from the C-terminal CBM fusion construct is consistent with the idea that perturbation of this mobile yet functionally important region compromises proper folding and/or conformational sampling. These results identify the C-terminal region as an engineering-sensitive element in GH134 enzymes and suggest that modifications at this site are poorly tolerated.

In contrast, fusion of SdCBM10 (the C-terminal CBM from *Saccharophagus degradans*) to the N terminus improved the apparent catalytic efficiency and thermal tolerance of AnGH134 toward locust bean gum, while also increasing tolerance under several chemically perturbed conditions under the assay conditions used. Because the N-terminal fusion does not interfere with the dynamic C-terminal region, it likely enhances substrate engagement without disrupting the conformational features required for catalysis. Structural comparison of the SdCBM10 and SsCBM10 models further suggests that SdCBM10 adopts a relatively compact architecture (Figure S6). This structural difference may reduce steric interference near the catalytic groove and may therefore be advantageous for substrate engagement in crowded, high-viscosity environments containing branched galactomannans. Such a compact architecture provides a plausible structural basis for the improved performance of the N-terminal fusion construct.

Overall, our results identify extended groove interactions and ligand-responsive local dynamics as key determinants of AnGH134 function. In particular, the C-terminal region appears to be both mechanistically relevant and sensitive to engineering perturbation, whereas N-terminal CBM fusion represents a promising strategy for improving catalytic efficiency, thermal robustness, and operational tolerance without disrupting the native catalytic framework. These findings expand the current understanding of GH134 mannanases and provide a useful structure-guided framework for future development of improved biocatalysts for mannan processing in food- and agricultural-relevant systems.

## Supplementary Material



## Notes

Table S1. Primers used in this study. Table S2. Protein engineering sequences of AnGH134 constructs. Figure S1. Purification and crystallization of recombinant AnGH134. Figure S2. Thermal stability and conformational analyses of wild-type AnGH134 and mutant variants. Figure S3. Expression and solubility analysis of the C-terminal fusion construct AnGH134-CBM. Figure S4. Michaelis–Menten kinetic analysis of AnGH134 and CBM-AnGH134 toward locust bean gum and ivory nut mannan. Figure S5. Structural snapshots of AnGH134 in apo and ligand-bound states during MD simulations. Figure S6. Structural comparison of SdCBM10 and SsCBM10 models (PDF)
