# Supplementary material for: Structural Characterization and Engineering of a GH134 β‑Mannanase from Aspergillus nidulans for Enhancement of Activity and Stability
Source: J Agric Food Chem. 2026 Aug 27;74(35):27687–97. doi: 10.1021/acs.jafc.6c05159 (PMC13573560; doi:10.1021/acs.jafc.6c05159)
Supplement: Supplementary file 1 [file jf6c05159_si_001.pdf]

Supplemental Information

Structural characterization and engineering of a GH134 β-Mannanase from *Aspergillus nidulans* for enhancement of activity and stability

Sheng-Chia Chen[1,2], Po-Chih Kuo[1], Wen-Ming Chen[2], Shih-Yi Sheu[3], Yu-Bin Huang[2], Cheng-Yang Huang[1], I-Weh Chien[1], Tzong-Huei Lee[4,5,6*], and Chun-Hua Hsu[1,7,8,9*]

[1]Department of Agricultural Chemistry, National Taiwan University, Taipei 106319, Taiwan.

[2]Department of Seafood Science, National Kaohsiung University of Science and Technology, Kaohsiung 811213, Taiwan

[3]Department of Marine Biotechnology, National Kaohsiung University of Science and Technology, Kaohsiung 811213, Taiwan

[4]Institute of Fisheries Sciences, National Taiwan University, Taipei 106319, Taiwan

[5]Department of Life Science, College of Life Science, National Taiwan University, Taipei 106319, Taiwan.

[6]Center for Biotechnology, National Taiwan University, Taipei 106319, Taiwan

[7]Institute of Biochemical Sciences, National Taiwan University, Taipei 106319, Taiwan.

[8]Genome and Systems Biology Degree Program, National Taiwan University and Academia Sinica, Taipei 106319, Taiwan.

[9]Center for Computational and Systems Biology, National Taiwan University, Taipei 106319, Taiwan.

*Corresponding author: Dr. Chun-Hua Hsu or Dr. Tzong-Huei Lee

E-mail: andyhsu@ntu.edu.tw, Tel: +886-2-3366-4468

Or Email: thlee1@ntu.edu.tw; Tel: +886-2-3366-1828

Table S1. Primers used in this study.

	**primer (F)**
CTR-F	5'- gcccatatggcgccgaccaccgatatgacc-3'
CTR-R	5'- cggctcgagttaaatcgcctgaacgtccacgatcgcttgaacatccaccca-3'
C-CBM1-R-1	5'-gatcgcttgaacgtccaccca-3'
C-CBM-F-2	5'- agcggtagcagcagcagcagcagc-3'
C-CBM-R-2	5'- ctcgagttattgaccgctgcaggt-3'
N-CBM-F-1	5'-gcccatatggcgacctgcaactggtac-3'
N-CBM-R-1	5'-gctgctgccaccgctgct-3'
N-CBM-F-2	5'-gcgccgaccaccgatatgaccaaa-3'
N-CBM-R-2	5'-ggcctcgagttagatcgcttgcacgtc-3'
Y15A-F	5'-gaccgctcacggtggcgctaccacggtccg-3'
Y15A-R	5'-cggaccgtggtagcgccaccgtgagcggtc-3'
W167A-F	5'- gcttgaacatccaccgcaaaacgggtatcgtcggttttgt-3'
W167A-R	5'-acaaaaccgacgatacccgttttgcggtggatgttcaagc-3'

Table S2. Protein engineering sequences of AnGH134 constructs

Protein	Sequence
AGH134-CBM	MAPTTDMTKRADRGSYTVSGLGQRKQAILNAGGNTLDLA IAMLETEGMTTDYTYGDGKTYDAANFGLFKQNWGMLRV CATRYGLAGQSEADWNNGAILNSNVYADVASRWDCQGY YGVDLWFAGHRNGASGLSNPNTDDINNYKSAVYWIQQQI DSNSVYKTDDTRFWVDVQAISGSSSSSSSSSTSSSSSSSSSS SSSGGSSATCNWYGTQYPMCTSTSSGWGWENNQSCISPST CSGQ
CBM-AnGH134	MGSSHHHHHHSSGLVPRGSHMATCNWYGTQYPMCTSTSS GWGWENNQSCISPSTCSGQSGSSSSSSSSSTSSSSSSSSSS SSGGSSAPTTDMTKRADRGSYTVSGLGQRKQAILNAGGNT LDLAIAMLETEGMTTDYTYGDGKTYDAANFGLFKQNWG MLRVCATRYGLAGQSEADWNNGAILNSNVYADVASRWD CQGYYGVDLWFAGHRNGASGLSNPNTDDINNYKSAVYWI QQQIDSNSVYKTDDTRFWVDVQAI

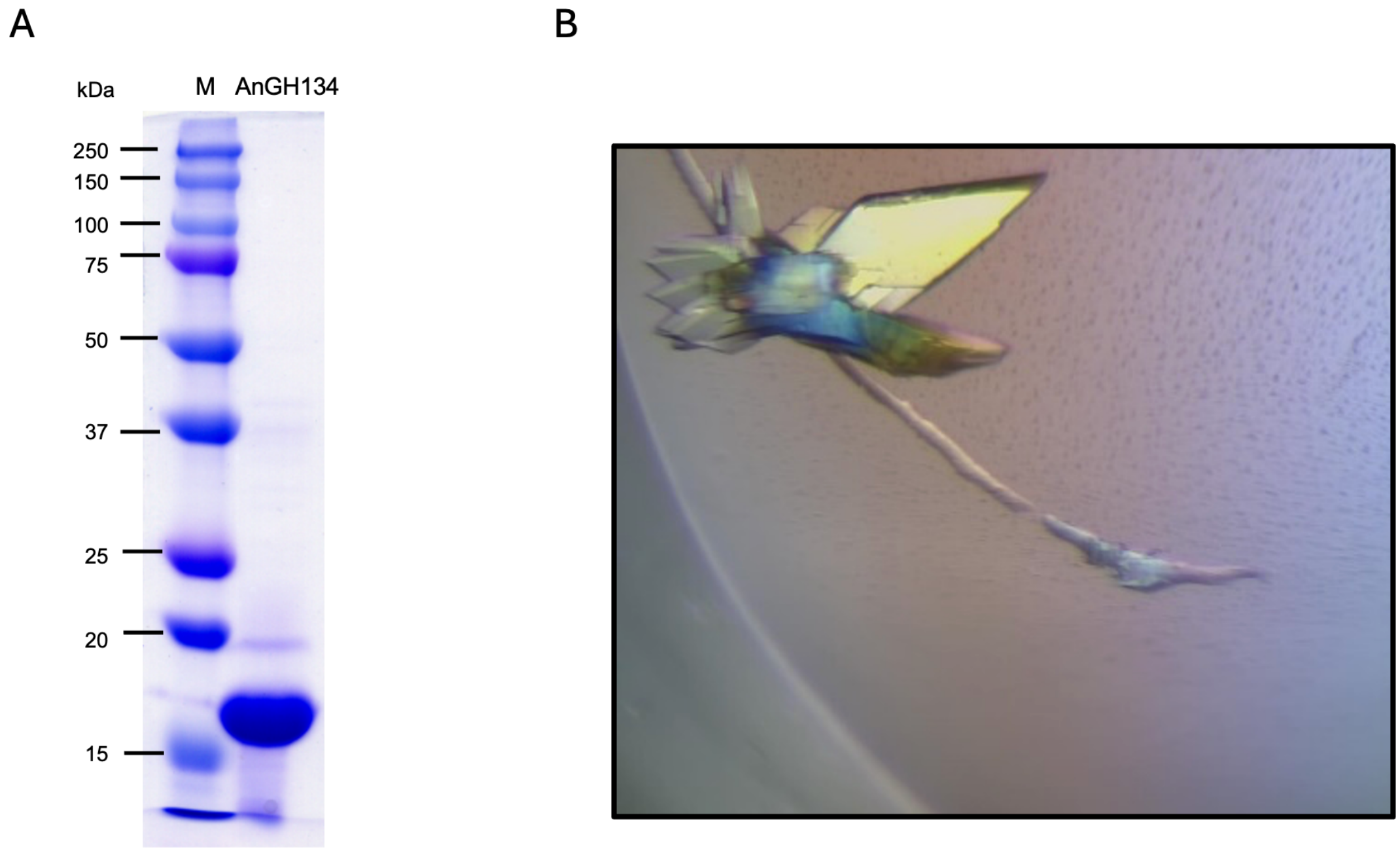

Figure S1. Purification and crystallization of recombinant AnGH134.

(A) SDS-PAGE analysis of purified recombinant AnGH134 after nickel-affinity chromatography and size-exclusion chromatography, showing that the protein was obtained with high purity. (B) Representative crystals of AnGH134 obtained under the optimized crystallization condition used for X-ray diffraction data collection.

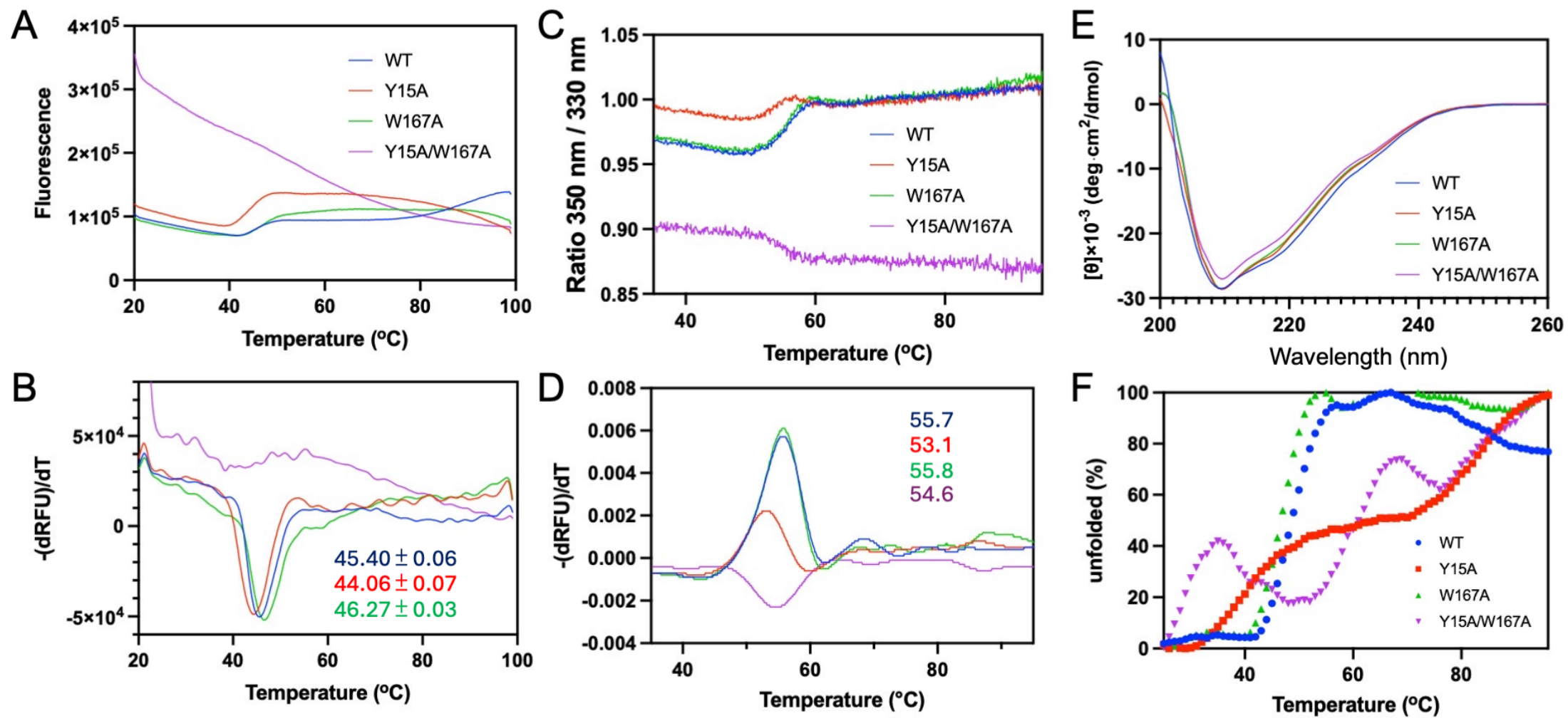

Figure S2. Thermal stability and conformational analyses of wild-type AnGH134 and mutant variants.

(A) Differential scanning fluorimetry (DSF) profiles of wild-type AnGH134 and the Y15A, W167A, and Y15A/W167A mutants. The double mutant did not exhibit a typical sigmoidal unfolding transition. (B) First-derivative curves of the DSF data shown in panel A. (C) nanoDSF unfolding profiles of wild-type AnGH134 and the corresponding mutants. (D) First-derivative curves of the nanoDSF data shown in panel C. (E) Far-UV CD spectra of wild-type AnGH134 and the mutant proteins. The Y15A/W167A double mutant displayed an altered CD spectrum relative to the wild-type enzyme. (F) Thermal denaturation monitored by CD at 209 nm. The Y15A and Y15A/W167A variants did not exhibit typical sigmoidal unfolding behavior under the conditions tested. Together, these data indicate that the single mutants show only modest differences in thermal stability by DSF/nanoDSF, whereas the double mutant displays more pronounced conformational perturbation.

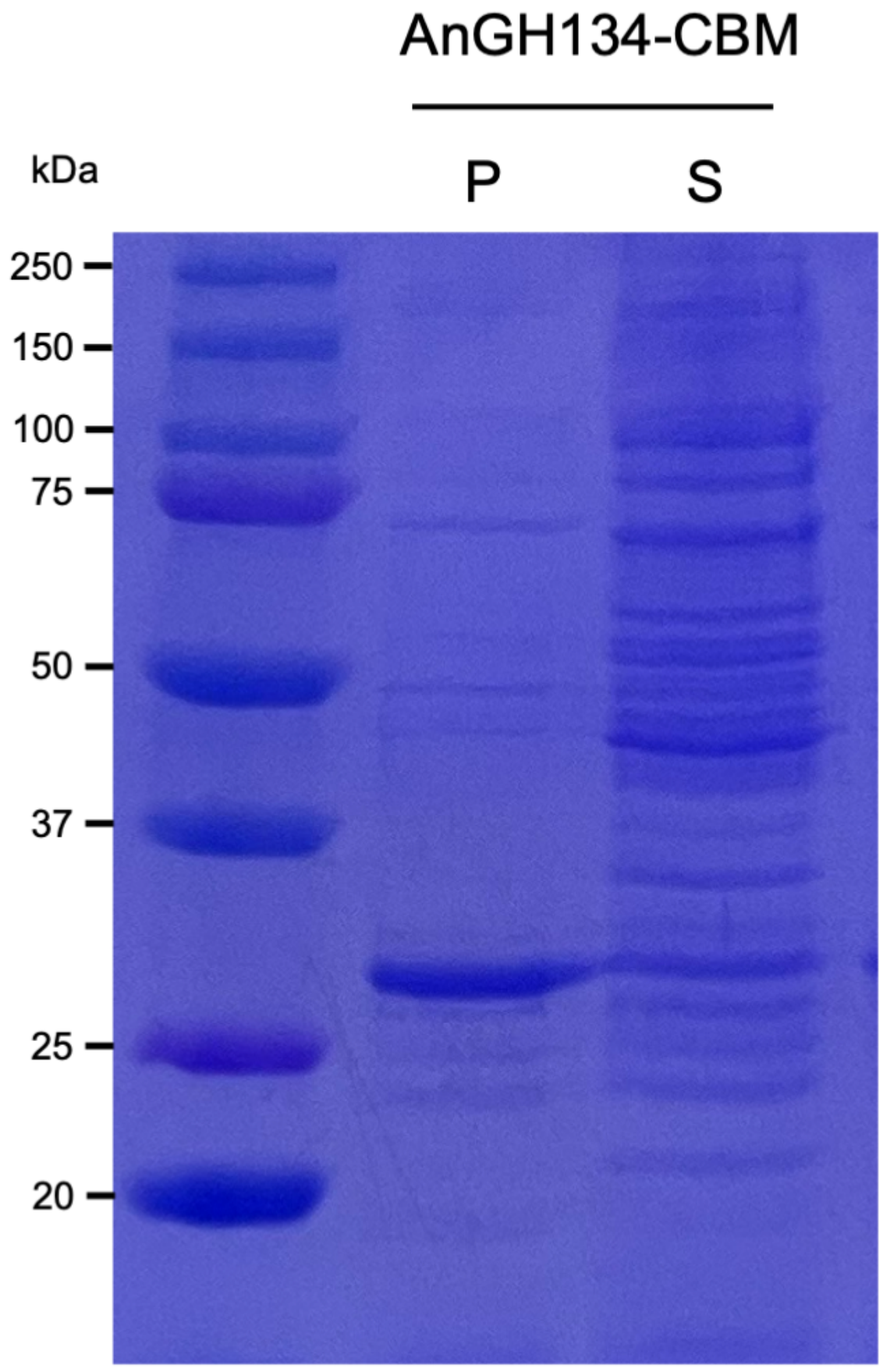

Figure S3. Expression and solubility analysis of the C-terminal fusion construct AnGH134-CBM.

SDS-PAGE analysis showing that the AnGH134-CBM fusion protein was expressed predominantly in the insoluble fraction (inclusion body) under the expression conditions used. No corresponding target protein was recovered from the soluble fraction during purification, preventing further biochemical characterization of this construct.

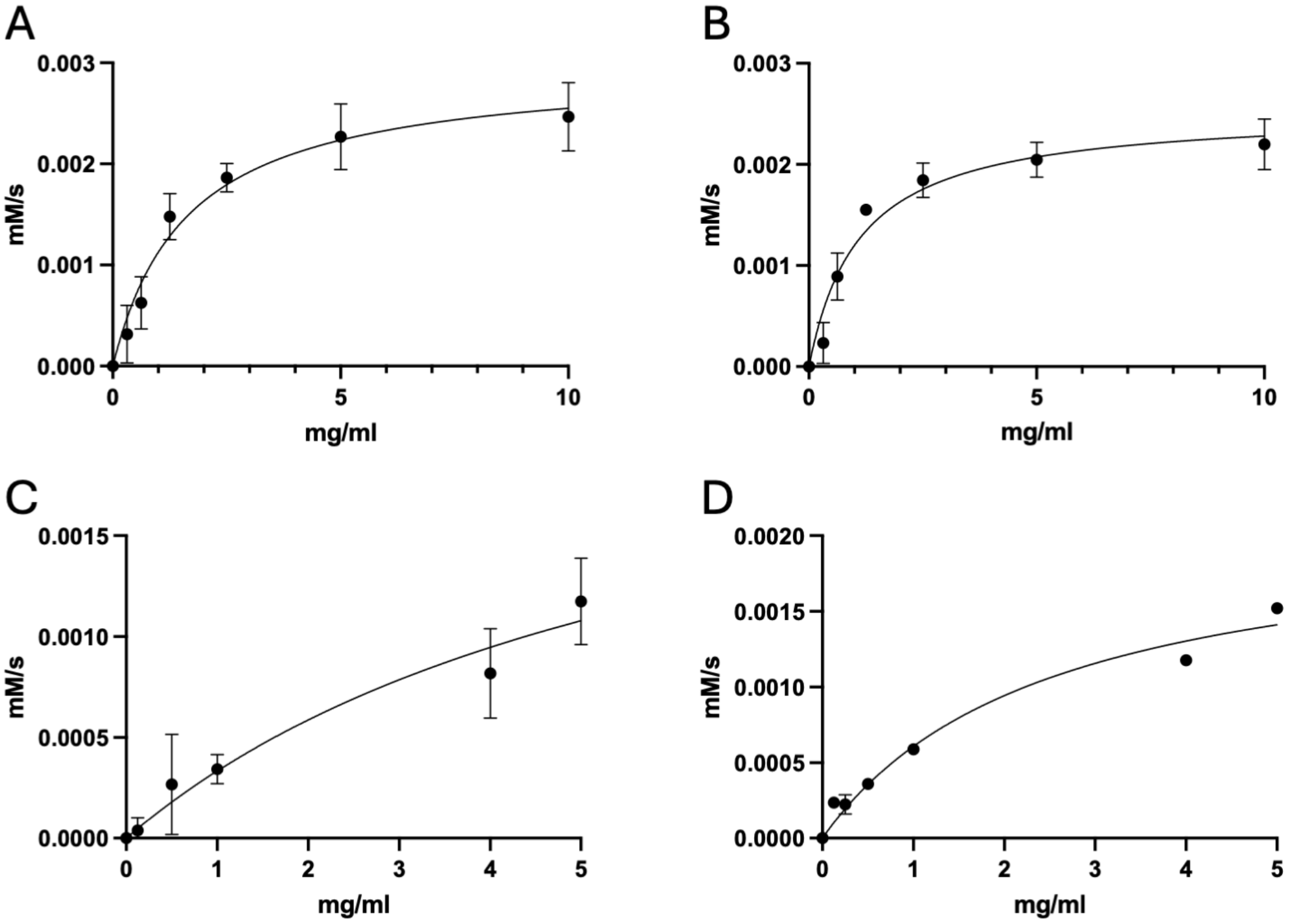

Figure S4. Michaelis–Menten kinetic analysis of AnGH134 and CBM-AnGH134 toward locust bean gum and ivory nut mannan.

(A) AnGH134 with LBG. (B) CBM-AnGH134 with LBG. (C) AnGH134 with INM. (D) CBM-AnGH134 with INM. The curves were fitted by nonlinear regression to the Michaelis–Menten equation using rates obtained from DNS-based reducing sugar assays. Because both substrates are heterogeneous polymers, the reported kinetic parameters represent apparent values for relative comparison.

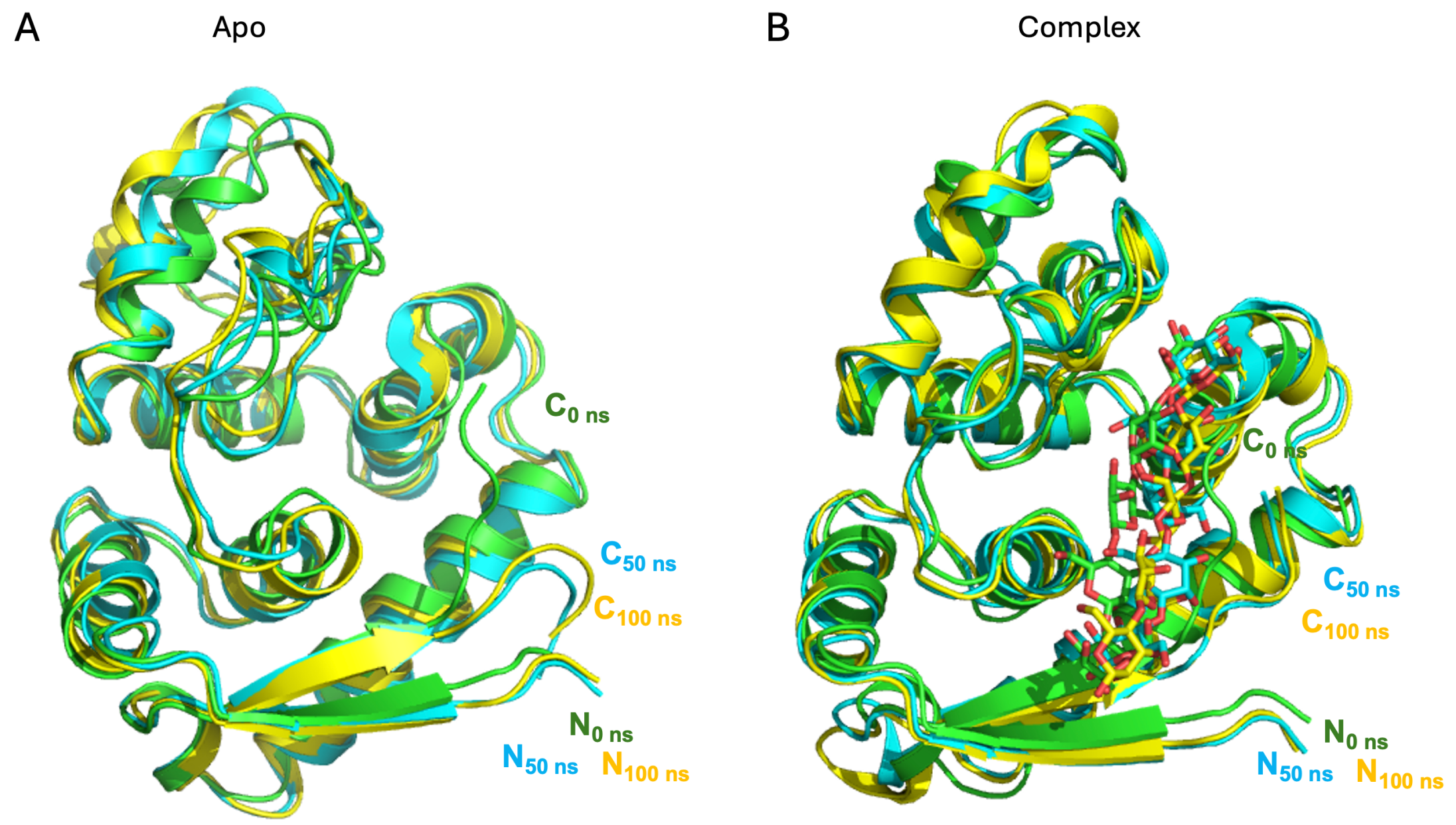

Figure S5. Structural snapshots of AnGH134 in apo and ligand-bound states during MD simulations.

Superposition of protein conformations at the beginning (0 ns, shown in cyan) and the end (200 ns, shown in yellow) of the molecular dynamics trajectories. (A) The left panel shows apo AnGH134 and highlights the intrinsic conformational flexibility of the enzyme in the absence of ligand. (B) The right panel shows the AnGH134-M5 complex and illustrates the reduced conformational deviation in the ligand-bound state, consistent with ligand-associated stabilization during the simulation.

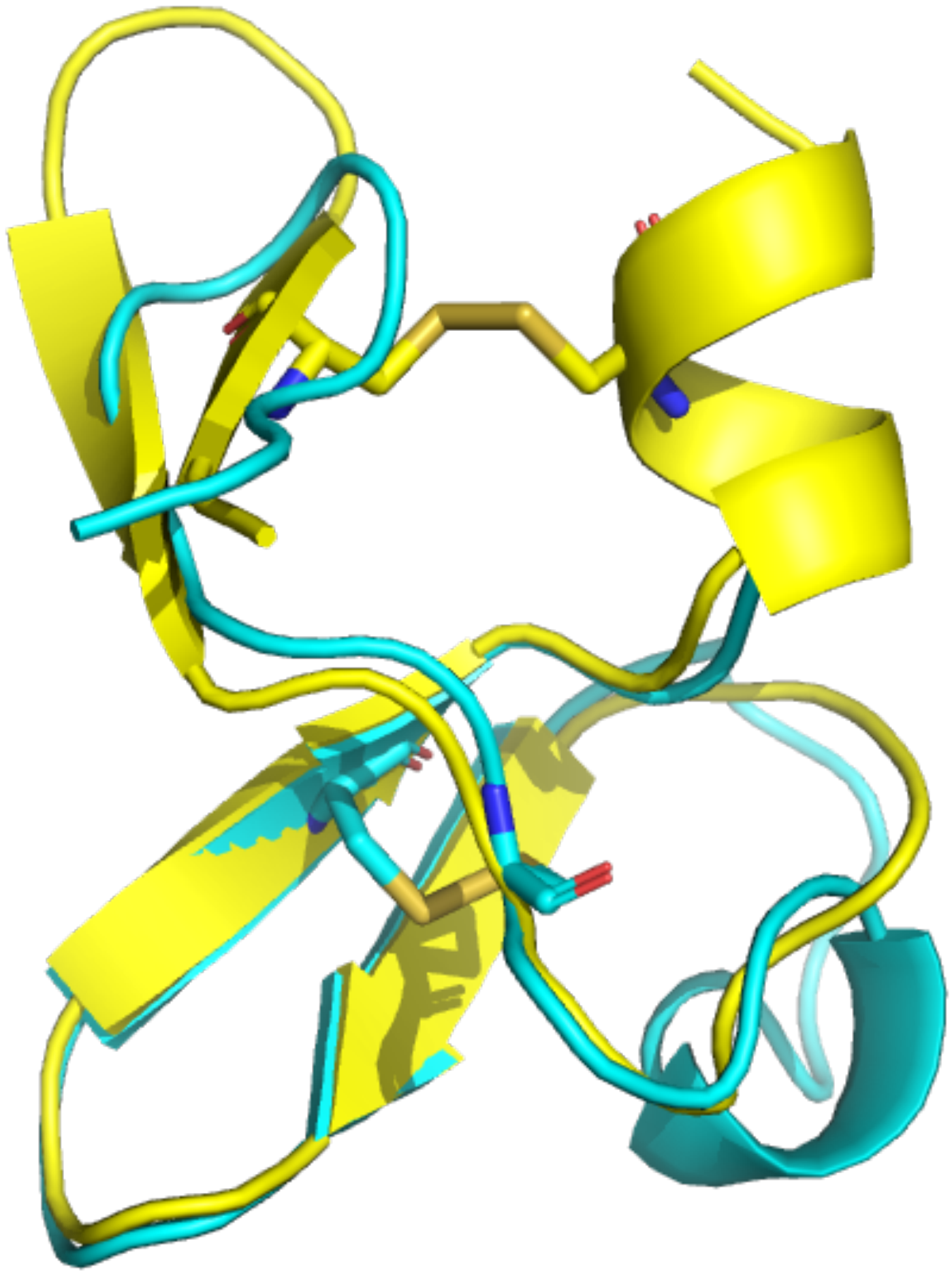

```
SdCBM10      ATCNWYGTQYPMCT------STSSGWGWENNQSCISPSTCSGQ     37
SsCBM10      --AETAPNGYPYCANGSASDPDGDGWGWENNRSCVVRTG----     37
               .:   . ** *:        ..*******:**:  :
```

Figure S6. Structural comparison of SdCBM10 and SsCBM10 models.

Structural superposition of the CBM10 module from *Saccharophagus degradans* (SdCBM10, yellow) and the CBM10 module from *Streptomyces* sp. NRRL B-24484 (SsCBM10, cyan). The comparison highlights the overall similarity of the CBM10 fold as well as local structural differences, including the more compact architecture of SdCBM10 relative to SsCBM10. Predicted disulfide bridges are shown as sticks.
